# Effective Permittivity of a Multi-Phase System: Nanoparticle-Doped Polymer-Dispersed Liquid Crystal Films

**DOI:** 10.3390/molecules26051441

**Published:** 2021-03-07

**Authors:** Doina Manaila-Maximean

**Affiliations:** Department of Physics, University Politehnica of Bucharest, Splaiul Independentei 313, 060042 Bucharest, Romania; doina.manaila@physics.pub.ro

**Keywords:** effective permittivity, liquid crystal, polymer-dispersed liquid crystal, nanoparticle

## Abstract

This paper studies the effective dielectric properties of heterogeneous materials of the type particle inclusions in a host medium, using the Maxwell Garnet and the Bruggeman theory. The results of the theories are applied at polymer-dispersed liquid crystal (PDLC) films, nanoparticles (NP)-doped LCs, and developed for NP-doped PDLC films. The effective permittivity of the composite was simulated at sufficiently high frequency, where the permittivity is constant, obtaining results on its dependency on the constituents’ permittivity and concentrations. The two models are compared and discussed. The method used for simulating the doped PDLC retains its general character and can be applied for other similar multiphase composites. The methods can be used to calculate the effective permittivity of a LC composite, or, in the case of a composite in which one of the phases has an unknown permittivity, to extract it from the measured composite permittivity. The obtained data are necessary in the design of the electrical circuits.

## 1. Introduction

Composite electro-optic materials, such as polymer/liquid crystal (LC) blends cover a large area of two phase mixtures as, for example: polymer-Ddspersed liquid crystal (PDLC) films [1], liquid crystal dispersed in an electrospun cellulose acetate network [2,3], cellulose film/LC composite [4], polymer balls/nematic LC films [5].

Polymer-dispersed liquid crystal (PDLC) films consist of micrometer or sub-micrometer-sized nematic droplets dispersed in a polymer matrix [6]. Their optical transmission response is based on the electrically controlled light scattering properties of the droplets. An applied electric field aligns the nematic droplets, and due to the refractive index match of the polymer and the aligned LC, a transparent non-scattering state is obtained. In the absence of the field, the molecules inside the droplets return to their original orientation and results in a scattering opaque state.

The reorientation of the LC molecules depends on the electric field across the droplet [6,7], and at a macroscopic scale, it depends on the dielectric permittivity. The LC droplets inside the polymer matrix form a bi-phase system, and to study the dielectric permittivity, the Maxwell Garnet [8] and the Bruggeman [9,10] effective medium models are considered in this paper. Generally, the permittivity has frequency dispersion, but conductivity effects become unimportant compared to dielectric effects at relatively high frequencies, where the ionic motion is frozen out, and at high resistivities, where the small number of mobile ions will not give rise to a significant depolarization field [1], and the present study considers this situation.

At present, many studies are dedicated to nanoparticles (NPs)-doped LCs [11] and PDLC films, in order to take advantage to each constituent beneficial contribution [12,13,14]. The contribution of the third phase formed by the dispersed NPs both in the LC droplets and in the polymer matrix is taken into account at the calculation of the effective dielectric permittivity of NPs-doped PDLC films.

It is generally accepted that the LC form droplets encapsulated in the polymer up till a concentration of about 50%. For greater concentrations, interconnecting channels will form between the LC droplets, the structure passing gradually in a sponge-like one.

Because LC doping with small fractions of NP is a very delicate process that might lead to tricky experimental results, it is important to use models to gain information about the effective dielectric constant of NPs containing multiphase systems.

## 2. Theoretical Models

The investigated medium is a heterogeneous composite at microscopic scale, where we can evaluate the effective dielectric function of the macroscopic uniform medium depending on the permittivity of the individual components and their respective volume fractions. Two of the most used effective medium approaches are the Maxwell Garnet [15,16] and the Bruggeman theories which are discussed further.

### 2.1. Maxwell Garnett (MG) Model

**Figure 1:**
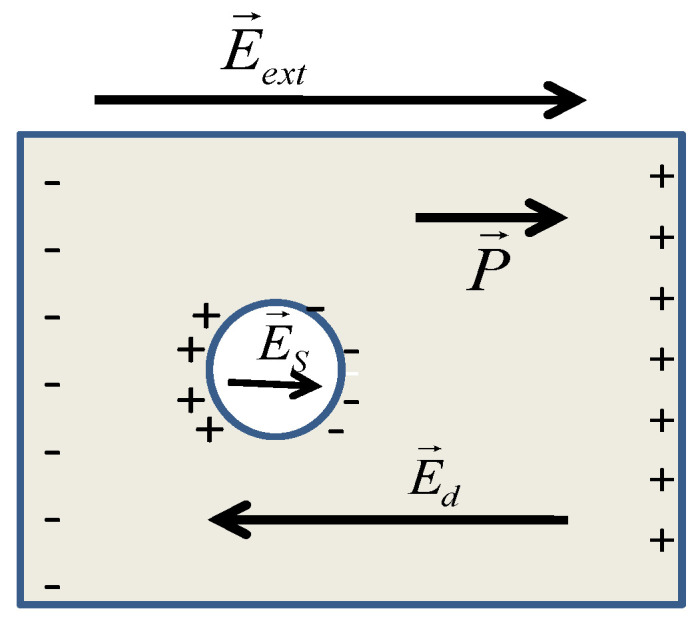
Auxiliary sphere around a molecule for determining the local electric field.

Let us consider a dense medium formed by molecular dipoles. Firstly, one should evaluate the local field at the site of a molecule, supposing that the molecule is surrounded by a spherical cavity of radius R, as seen in Figure 1. The space inside the sphere has free space permittivity, since it is the space between two molecules. When applying an external electric field ${\vec{E}}_{ext}$, the electrical charges will move according to their sign, producing the electric field ${\vec{E}}_{S}$ due to the polarization charges, which, in the case of the considered sphere, is $E_{S}=\frac{P}{3\varepsilon_{0}}$, where *P* is the macroscopic polarization. The local field acting in the central of the dipole is

(1) $${\vec{E}}_{L}={\vec{E}}_{ext}+{\vec{E}}_{S}+{\vec{E}}_{d}+{\vec{E}}_{near}$$

where ${\vec{E}}_{d}$ is the depolarization field lying at the external surface of the medium, $E_{d}=\frac{-P}{\varepsilon_{0}}$; ${\vec{E}}_{near}$ is the field induced by other dipoles lying within the sphere, which, in the case of a symmetric cubic lattice, vanishes. Since the sum ${\vec{E}}_{ext}+{\vec{E}}_{d}=\vec{E}$, where $\vec{E}$ is the macroscopic electric field, the local field becomes:

(2) $${\vec{E}}_{L}=\vec{E}+\frac{\vec{P}}{3\varepsilon_{0}}$$

(3) $$P=N\alpha E_{L}=N\alpha \left(E+\frac{P}{3\varepsilon_{0}}\right)$$

where α is the polarizability of one molecule and *N* is the volume density of dipoles.

The electric induction being

(4) $$\vec{D}=\varepsilon_{0}\vec{E}+\vec{P}=\varepsilon_{0}\varepsilon_{r}\vec{E}$$

Using Equations’ (3) and (4) results, the Clausius–Mossotti relation is

(5) $$\frac{N\alpha }{3\varepsilon_{0}}=\frac{\varepsilon_{r}-1}{\varepsilon_{r}+2}$$

Considering the Clausius–Mossotti relation for a composite formed of spherical particles of relative permittivity $\varepsilon_{1}$ embedded in a host medium of relative permittivity $\varepsilon_{h}$:

(6) $$\frac{N\alpha }{3\varepsilon_{0}\varepsilon_{h}}=\frac{\varepsilon_{eff,MG}-\varepsilon_{h}}{\varepsilon_{eff,MG}+2\varepsilon_{h}}$$

where $\varepsilon_{eff,MG}$ is the effective permittivity obtained using the MG formula. Considering *f* the volume filling factor of the spheres, the polarizability in relation (5) becomes:

(7) $$\alpha =\frac{3\varepsilon_{0}\varepsilon_{h}f}{N}\frac{\varepsilon_{1}-\varepsilon_{h}}{\varepsilon_{1}+2\varepsilon_{h}}$$

By substituting Equation (7) in (6), the MG formula is obtained:

(8) $$\frac{\varepsilon_{eff,MG}-\varepsilon_{h}}{\varepsilon_{eff,MG}+2\varepsilon_{h}}=f\frac{\varepsilon_{1}-\varepsilon_{h}}{\varepsilon_{1}+\varepsilon_{h}}$$

(9) $$\varepsilon_{eff,MG}=\varepsilon_{h}\frac{1+2f\frac{\varepsilon_{1}-\varepsilon_{h}}{\varepsilon_{1}+2\varepsilon_{h}}}{1-f\frac{\varepsilon_{1}-\varepsilon_{h}}{\varepsilon_{1}+2\varepsilon_{h}}}$$

To model the capacity properties of the pure PDLC, we have chosen the MG formula adapted for the LC-polymer bi-phase system:

(10) $$\varepsilon_{eff,MG}=\varepsilon_{ply}\frac{1+2f_{LC}\frac{\varepsilon_{LC}-\varepsilon_{ply}}{\varepsilon_{LC}+2\varepsilon_{ply}}}{1-f_{LC}\frac{\varepsilon_{LC}-\varepsilon_{ply}}{\varepsilon_{LC}+2\varepsilon_{ply}}}$$

where $\varepsilon_{eff,MG}$ is the effective dielectric constant of the composite PDLC film, $\varepsilon_{ply}$ is the dielectric constant of the polymeric matrix, $\varepsilon_{LC}$ is the dielectric constant of the dispersed liquid crystal, and $f_{LC}$ is the volume fraction of the LC.

LCs are anisotropic materials and the dielectric anisotropy in the uniaxial nematic phase is characterized by two principal components, one component is parallel, $\varepsilon_{II}$, and the other component is perpendicular $\varepsilon_{⊥}$ to the director of the LC. The dielectric anisotropy Δε in the uniaxial phases is $\Delta \varepsilon =\varepsilon_{II}-\varepsilon_{⊥}$ and its sign depends on the chemical structure of the constituent molecules [17]. The average permittivity of the LC is defined as $\varepsilon_{LC}=\varepsilon_{LC,random}=\frac{\varepsilon_{II}+2\varepsilon_{⊥}}{3}$ and is used in our discussion. By measuring the capacitance of the film, one can determine an effective permittivity in zero-applied electric field ($\varepsilon_{⊥}$) and in the high-applied electric field (corresponding to the oriented LC, and to $\varepsilon_{II}$) states. Equation (9) can be applied to obtain the effective permittivity of the film (corresponding to $\varepsilon_{⊥}$, $\varepsilon_{II}$, and $\varepsilon_{LC,random}$) and the results are presented in Section 4.

### 2.2. Bruggeman Effective Medium Model 

The model considers spherical particles of two different materials, of permittivities $\varepsilon_{1}$, $\varepsilon_{2}$ dispersed in a host matrix of dielectric constant $\varepsilon_{h}$ [9,15]. For a two inclusion composite, induced in a symmetric manner, the following expression was obtained:

(11) $$\frac{\varepsilon_{eff,B}-\varepsilon_{h}}{\varepsilon_{eff,B}+2\varepsilon_{h}}=f_{1}\frac{\varepsilon_{1}-\varepsilon_{h}}{\varepsilon_{1}+2\varepsilon_{h}}+f_{2}\frac{\varepsilon_{2}-\varepsilon_{h}}{\varepsilon_{2}+2\varepsilon_{h}}$$

where $\varepsilon_{eff,B}$ is the Bruggeman’s effective permittivity of the medium.

For a two phase system, $f_{1}+f_{2}=1$, if each material is considered as one inclusion and the host medium is the material itself. In this case $\varepsilon_{eff,B}=\varepsilon_{\begin{array}{l} h \end{array}}$ and the left hand side of Equation (6) becomes zero. It follows:

(12) $$f_{1}\frac{\varepsilon_{1}-\varepsilon_{eff,B}}{\varepsilon_{1}+2\varepsilon_{eff,B}}+f_{2}\frac{\varepsilon_{2}-\varepsilon_{eff,B}}{\begin{array}{l} \varepsilon_{2}+2\varepsilon_{eff,B} \end{array}}=0$$

The solution of Equation (12) is

(13) $$\varepsilon_{eff,B}=\frac{1}{4}\{\left(3f_{1}-1\right)\varepsilon_{1}+\left(3f_{2}-1\right)\varepsilon_{2}\pm \sqrt{{\left[\left(3f_{1}-1\right)\varepsilon_{1}+\left(3f_{2}-1\right)\varepsilon_{2}\right]}^{2}+8\varepsilon_{1}\varepsilon_{2}}\}$$

The sign in Equation (13) is chosen such that the imaginary part of the effective permittivity is positive. For the pure PDLC, the effective permittivity considering the Bruggeman expression, $\varepsilon_{eff,B}$ becomes:

(14) $$\varepsilon_{eff,B}=\frac{1}{4}\left\{\left(3f_{LC}-1\right)\varepsilon_{LC}+\left(3f_{ply}-1\right)\varepsilon_{ply}\pm \sqrt{{\left[\left(3f_{LC}-1\right)\varepsilon_{LC}+\left(3f_{ply}-1\right)\varepsilon_{ply}\right]}^{2}+8\varepsilon_{LC}\varepsilon_{ply}}\right\}$$

### 2.3. NPs-Doped LC

The LC droplet is doped with NPs and the effective permittivity of the doped LC considering the MG formula is (15), resulted from (10), and considering the Bruggeman expression is presented in (16) (resulted from (14)), respectively.

(15) $$\varepsilon_{LC\_NP,MG}=\varepsilon_{LC}\frac{1+2f_{NP}\frac{\varepsilon_{NP}-\varepsilon_{LC}}{\varepsilon_{NP}+2\varepsilon_{LC}}}{1-f_{NP}\frac{\varepsilon_{NP}-\varepsilon_{LC}}{\varepsilon_{NP}+2\varepsilon_{LC}}}$$

(16) $$\varepsilon_{LC\_NP,B}=\frac{1}{4}\left\{\left(3f_{NP}-1\right)\varepsilon_{NP}+\left(3\left(1-f_{NP}\right)-1\right)\varepsilon_{LC}\pm \sqrt{{\left[\left(3\left(1-f_{NP}\right)-1\right)\varepsilon_{LC}+\left(3f_{NP}-1\right)\varepsilon_{NP}\right]}^{2}+8\varepsilon_{LC}\varepsilon_{NP}}\right\}$$

where we have taken into consideration that the sum between the NPs volume fraction (in LC) and the LC volume fraction (in NPs) is 1.

### 2.4. NPs-Doped PDLC Films

**Figure 2:**
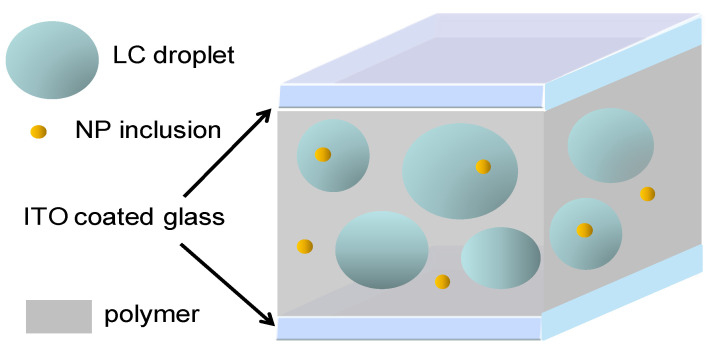
Schematic presentation of a nanoparticles (NPs) polymer-dispersed liquid crystal (PDLC)-doped film between two indium tin oxide (ITO) glass coated plates: LC droplets are spherical.

To obtain the effective permittivity for the doped PDLC, the following system is considered: the NPs-doped polymer matrix, LC droplets doped with NPs, as presented in Figure 2.

Since the fraction of NPs in LC is very small, we may consider the MG formula for the NPs doped polymer, and using Equation (10) it follows:

(17) $$\varepsilon_{ply,NP\_MG}=\varepsilon_{ply}\frac{1+2f_{NP}\frac{\varepsilon_{NP}-\varepsilon_{ply}}{\varepsilon_{NP}+2\varepsilon_{ply}}}{1-f_{NP}\frac{\varepsilon_{NP}-\varepsilon_{ply}}{\varepsilon_{NP}+2\varepsilon_{ply}}}$$

For the doped LC, we consider Equation (14).

Finally, for the NPs-doped PDLC, we use the Bruggeman formula:

(18) $$\varepsilon_{PDLC,NP}=\frac{1}{4}\left\{\left(3f_{LC}-1\right)\varepsilon_{LC\_NP,MG}+\left(3\left(1-f_{LC}\right)-1\right)\varepsilon_{ply\_NP,MG}\pm \sqrt{\Delta }\right\}$$

(19) $$\Delta ={\left[\left(3\left(1-f_{LC}\right)-1\right)\varepsilon_{ply\_NP,MG}+\left(3f_{LC}-1\right)\varepsilon_{LC\_NP,MG}\right]}^{2}+8\varepsilon_{LC\_NP,MG}\varepsilon_{ply\_NP,MG}$$

## 3. Results and Discussions

### 3.1. PDLC Film

**Figure 3:**
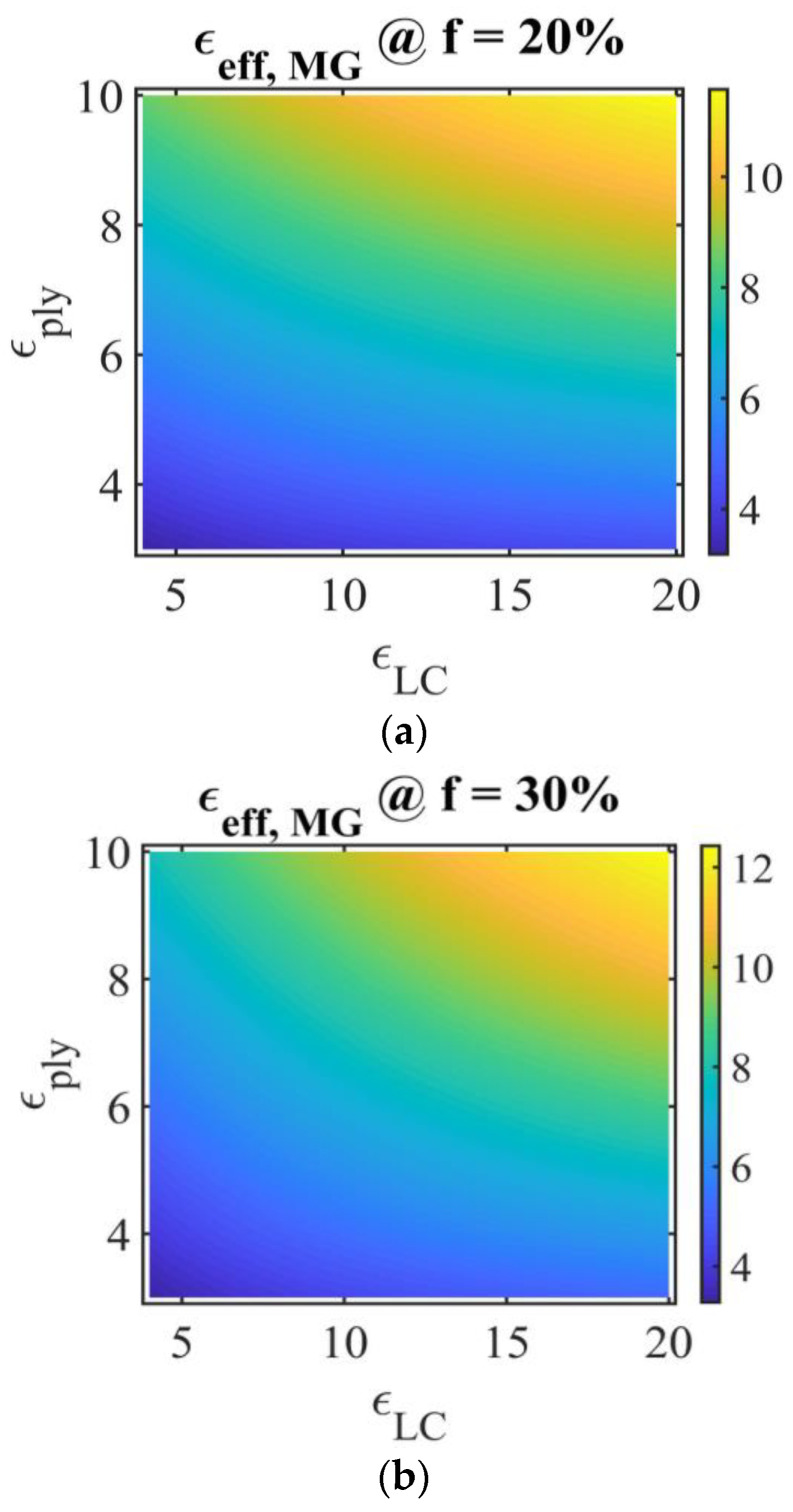

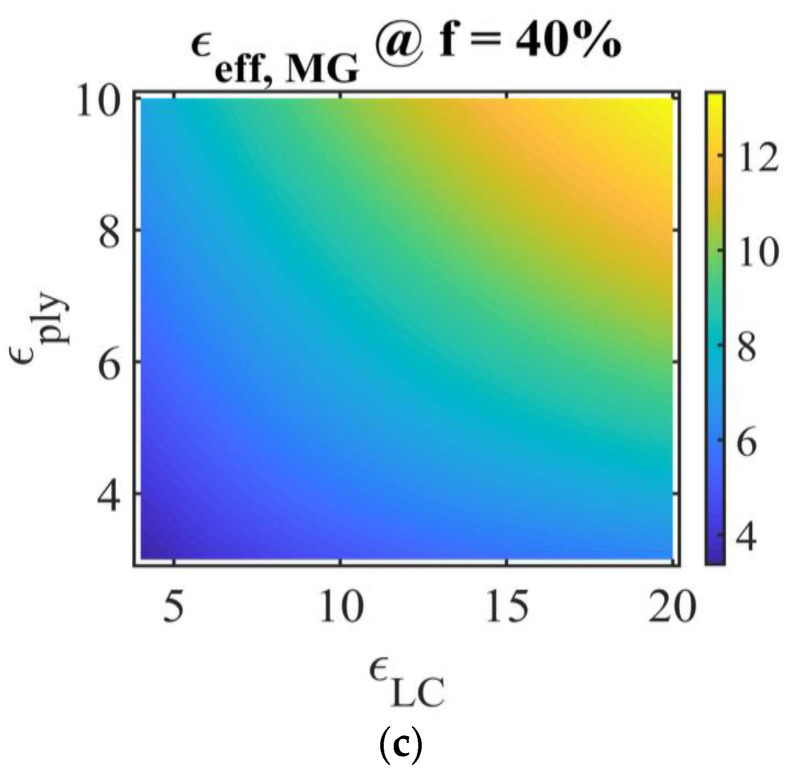
Representation of PDLC permittivity in the Maxwell Garnett model, for different LC fractions: (**a**) *f_LC,a_* = 0.2; (**b**) *f_LC,b_ =* 0.3; (**c**) *f_LC,c_* = 0.4. Ox: LC permittivity $\varepsilon_{LC}$, Oy: polymer permittivity $\varepsilon_{ply}$; vertical color bar: PDLC film effective permittivity using the Maxwell Garnet model, symbol @ stands for “at”.

Figure 3 shows the numerical representation of PDLC effective permittivity, vertical colour bar, Ox axis represents LC permittivity $\varepsilon_{LC}$, Oy axis represents polymer permittivity $\varepsilon_{ply}$, obtained using the Maxwell Garnett model (Equation (10)), for LC volume fractions of 20%, 30%, and 40%, commonly used in the study of PDLC films and for whom LC forms individual droplets. The numerical values in the following discussions refer to the relative permittivity of the materials. The polymer permittivity variation domain is chosen between 3 and 10, and the LC permittivity between 4 and 20, considering their values at high frequency [17,18,19,20]. As easily seen in Figure 3a, at constant volume fraction and constant LC permittivity, the effective MG permittivity increases with the polymer permittivity, and at constant polymer permittivity, the effective MG permittivity increases with the LC permittivity.

**Figure 4:**
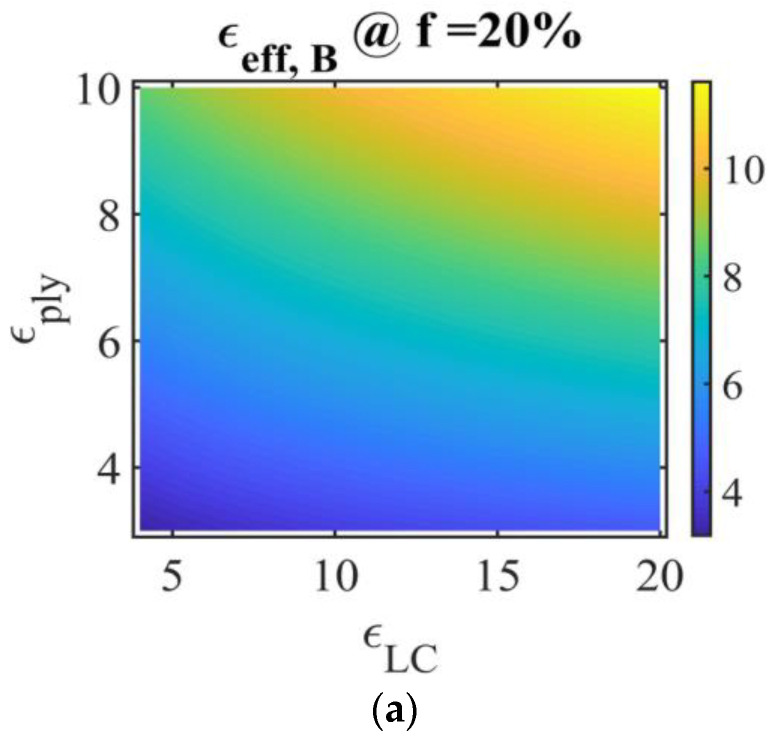

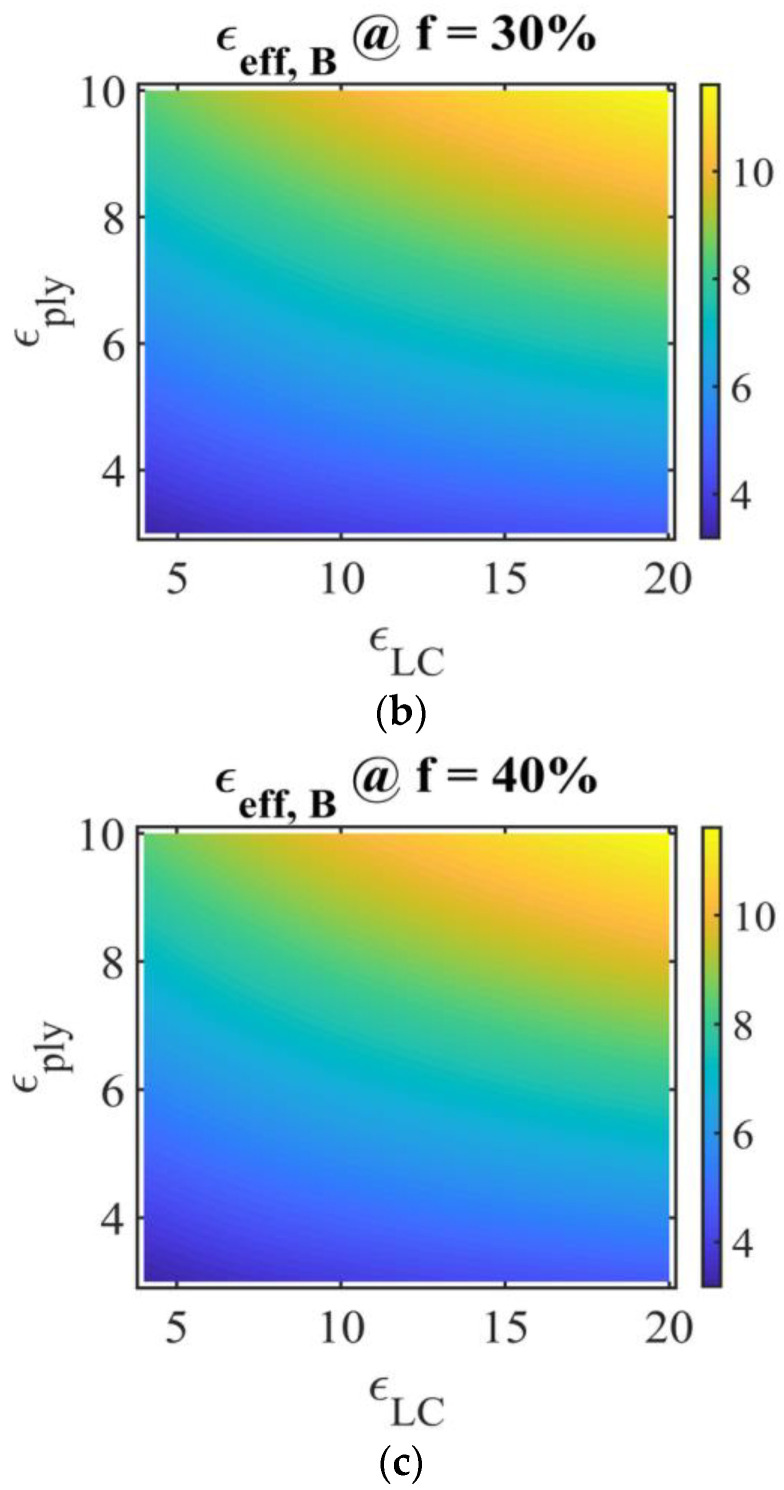
Representation of PDLC film effective permittivity in the Bruggeman model for different LC fractions: (**a**) $f_{LC,a}=0.2$, (**b**) $f_{LC,b}=0.3$, (**c**) $f_{LC,c}=0.4$. Ox: LC permittivity $\varepsilon_{LC}$, Oy: polymer permittivity $\varepsilon_{ply}$; vertical color bar: PDLC film effective permittivity using the Bruggeman model, symbol @ stands for “at”.

In Figure 4, the numerical simulations of PDLC effective permittivity (vertical colour bar) are presented, obtained using the Bruggeman model (Equation (14)), for LC volume fractions of 20%, 30%, and 40%, Ox axis: LC permittivity $\varepsilon_{LC}$, Oy axis: polymer permittivity $\varepsilon_{ply}$. At constant LC permittivity, the effective PDLC permittivity increases with the polymer permittivity. Considering, for example, the lower limit for the LC permittivity and the upper limit for the polymer permittivity, $\varepsilon_{LC}=4$ and $\varepsilon_{ply}=10$, the effective Bruggeman permittivity for the PDLC film decreases with the increase of the LC fraction.

**Figure 5:**
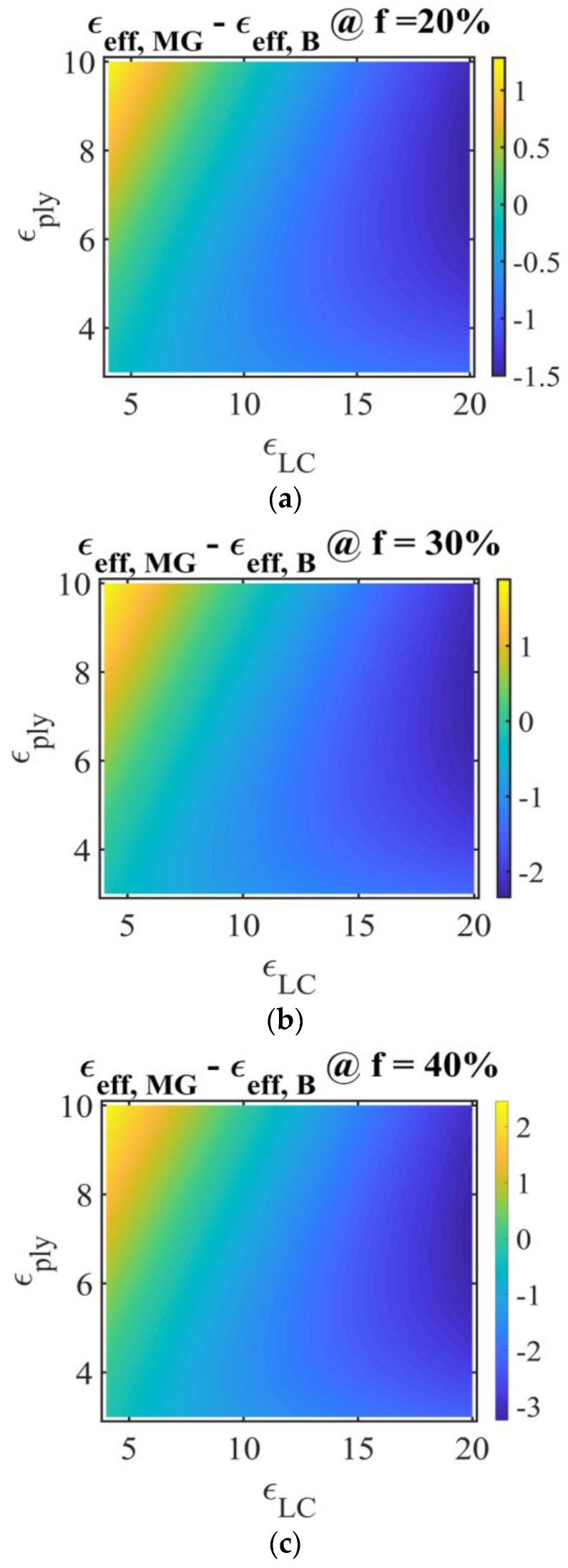
The difference between the Maxwell Garnett (MG) and Bruggeman effective permittivity, $\varepsilon_{eff,MG}-\varepsilon_{eff,B}$, (**a**) *f_LC,a_* = 0.2; (**b**) *f_LC,b_ =* 0.3; (**c**) *f_LC,c_* = 0.4.. Ox: LC permittivity $\varepsilon_{LC}$, Oy: polymer permittivity $\varepsilon_{ply}$; vertical color bar: the difference between the MG and Bruggeman effective permittivity of the PDLC film, $\varepsilon_{eff,MG}-\varepsilon_{eff,B}$ symbol @ stands for “at”.

To compare the results predicted by the two models, Figure 5 presents the difference between the MG and Bruggeman effective permittivity, $\varepsilon_{eff,MG}-\varepsilon_{eff,B}$, for three LC fractions. Considering, for example, the LC fraction of 20%, $\varepsilon_{ply}=3$, $\varepsilon_{LC}=10$, it results in an effective Bruggeman permittivity greater than the effective MG one. At $\varepsilon_{ply}=10$ and $\varepsilon_{LC}=5$, the MG effective permittivity is greater than the Bruggeman one. The modulus $\left|\varepsilon_{eff,MG}-\varepsilon_{eff,B}\right|$ increases with the LC fraction, at constant constituents’ permittivity.

### 3.2. NPs-Doped LCs

**Figure 6:**
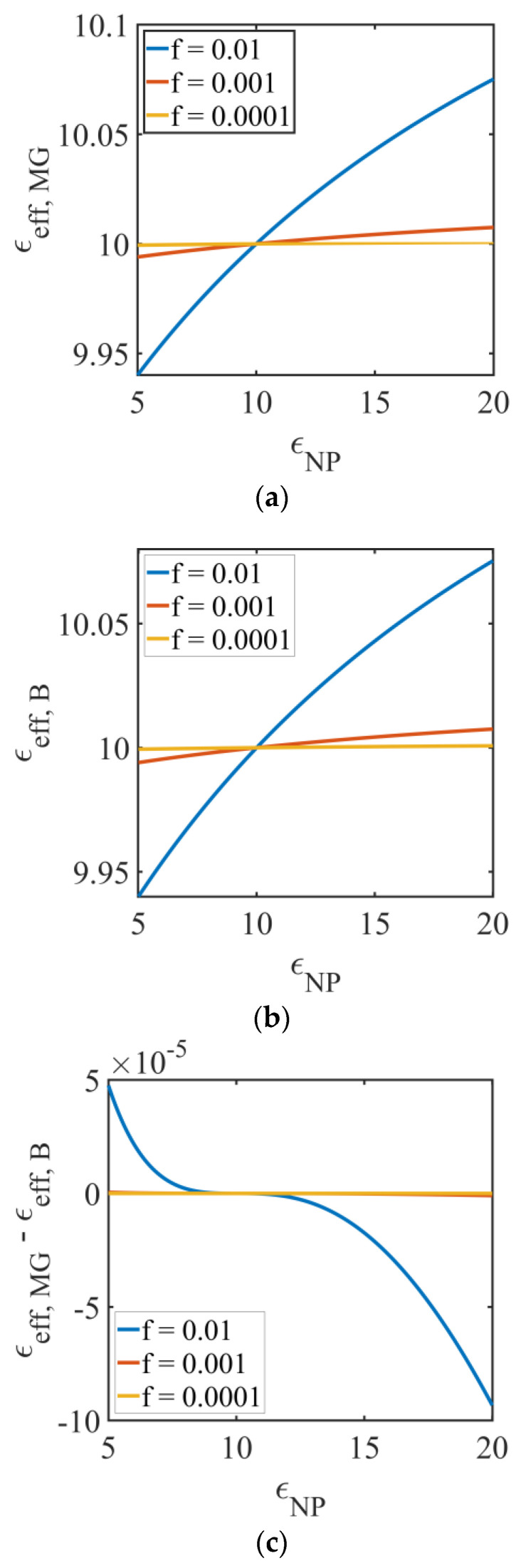
Representation of effective permittivity for NPs-doped LC versus NP permittivity, $\varepsilon_{NP}$, for three NP volume fractions: 0.01; 0.001; and 0.0001, (**a**) MG model (Equation (14)); (**b**) Bruggeman model (Equation (15)), and (**c**) the difference of the effective permittivity obtained using these models.

Figure 6 presents the effective permittivity for NPs-doped LC versus NPs’ permittivity, $\varepsilon_{NP}$, for three NP volume fractions: 0.01; 0.001; and 0.0001, using the MG model (Equation (15), Figure 6a) and the Bruggeman model (Equation (16) Figure 6b). The LC permittivity is considered 10. In both cases, the effective permittivity increases with $\varepsilon_{NP}$ and with the NP volume fraction, excepting the case $\varepsilon_{NP}=\varepsilon_{LC}$ (in our case equal 10), when the volume fraction of NP does not influence the result (intersection of the three curves corresponding to the difference of NP volume fractions).

Comparing the two models, Figure 6c shows the difference $\varepsilon_{eff,MG}-\varepsilon_{eff,B}$ for the NPs-doped LCs. It results that $\varepsilon_{eff,MG}>\varepsilon_{eff,B}$, when $\varepsilon_{NP}<10$ (which corresponds to $\varepsilon_{NP}=\varepsilon_{LC}$) and then it changes its sign, the difference being noticeable at NP volume fraction of 0.01, but still of the order of $10^{-4}$.

**Figure 7:**
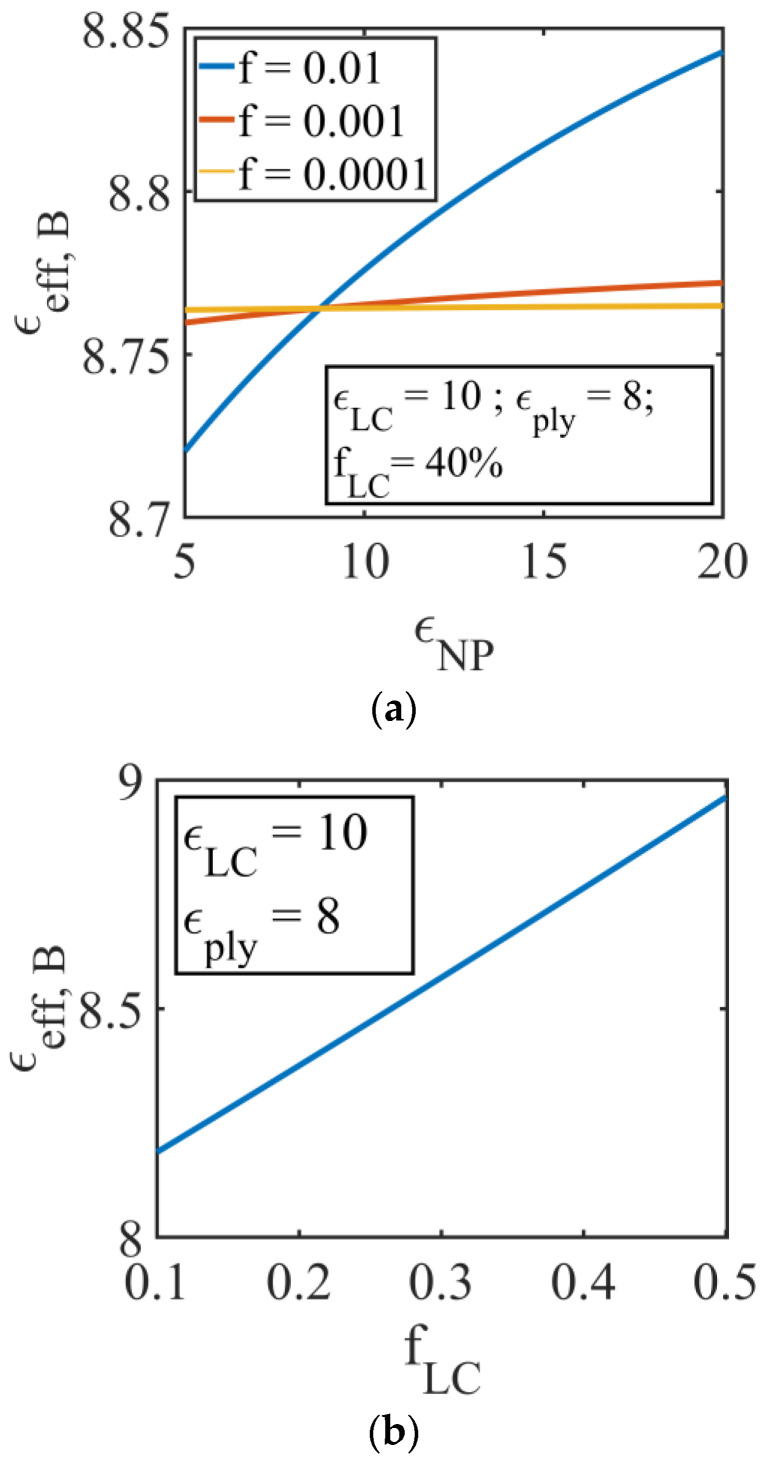
(**a**) Effective permittivity for the NPs-doped PDLC, versus NP permittivity, $\varepsilon_{NP}$, (Equation (18)) at a constant LC volume fraction $f_{LC}=0.4$ and for different NP volume fractions $f_{NP}$: 0.01; 0.001; and 0.0001. LC permittivty $\varepsilon_{LC}=10$, polymer permittivity $\varepsilon_{ply}=8$; (**b**) representation of undoped PDLC effective permittivity in the Bruggeman model versus LC volume fractions, equation (14), at constant LC permittivty $\varepsilon_{LC}=10$, polymer permittivity $\varepsilon_{ply}=8$.

Figure 7a represents the effective permittivity for the NPs-doped PDLC (Equation (17)), versus $\varepsilon_{NP}$, at a constant LC volume fraction $f_{LC}=0.4$ and for three NP volume fractions $f_{NP}$: 0.01; 0.001; and 0.0001. For the LC permittivity, the value of a commercial LC mixture, E7, was chosen at high frequency [18] $\varepsilon_{LC}=10$, and for the polymer, the permittivity of polyvinyl alcohol (PVA), $\varepsilon_{ply}=8$ [21,22,23,24,25]. The effective permittivity shows a modest increase at very low NP concentrations, and a more significant variation at $f_{Np}=0.01$. Figure 7b shows the effective Bruggeman permittivity versus LC concentration, for the undoped PDLC film.

When comparing the experimental values with the simulated ones, differences might appear due to the errors in the determination of the geometrical dimensions of the sample. To avoid this problem, in many dielectric spectroscopy studies, the aim is to obtain the characteristic time of the dielectric relaxation processes, independent on the sample’s dimension [10,11].

## 4. Comparison of Model Predictions with Test Data for PDLC Films

In order to compare the methods presented with experimental data from the literature [21,22,23,24,25], we chose a PDLC film consisting of the polyvinyl alcohol (PVA) polymer and nematic LCs [25]. The volume fraction is $f_{1}=f_{2}=0.5$. The dielectric constant of PVA is 8. Table 1 presents the permittivity of the LC (according to the producers’ data sheet [25]) and the LC used for the calculation of the effective permittivity to test the Maxwell Garnett (Equation (10)) and Bruggeman (Equation (14)) models. The effective permittivity of the PDLC film is calculated considering the LC permittivity, each of the corresponding values for $\varepsilon_{⊥}$, $\varepsilon_{II}$ and $\varepsilon_{LC,random}$. The dielectric constant of the PDLC film was measured in [25], in the “rest” states (corresponding to $\varepsilon_{⊥}$), zero electric field permittivity, and in the field-aligned states (corresponding to $\varepsilon_{II}$) high electric field permittivity.

The relative deviation of the calculated permittivity values is small. The calculated values in these particular cases show that the two models are correct, in agreement with the experiment, and with the ones calculated in [25] and suitable for the calculation of the composites’ permittivity [24,25]. The methods can be used to calculate the effective permittivity of a LC composite. If a series of LC composites are studied, measurements can be done only on some particular ones and the other can be calculated. In the case of a composite in which one of the phases has an unknown permittivity, this can be obtained by measuring the permittivity of the composite and using one of the models presented. The effective permittivity is necessary in the design of the electrical circuits where these opto-electronic materials are applied.

## 5. Conclusions

LC composites have a great importance in applications and are the subject of numerous theoretical studies. Knowing their permittivity helps us to calculate the electric field at which the reorientation effects of LC molecules occur, which gives rise to the desired electro-optical effects.

Unlike other models of permittivity calculation (volumetric models—linear or logarithmic [18,26]), MG and Bruggeman models have a theoretical basis, by virtue of their derivation from electrical principles.

PDLC being composites containing LC droplets in polymer form and a biphasic system for which the MG and Bruggeman mixing models can be used. This paper applies, develops, and compares the two models by mixing the permittivity of the constituents to obtain the effective permittivity of the material. NP doping of LCs and PDLC films is a current research topic of great interest. The MG and Bruggeman models were also applied in case of NP doping of LCs.

To study NP-doped PDLCs, given the very low concentrations used in doping, we used the MG model to obtain two biphasic media, one consisting of NP in LC and the other of NP in polymer. The three-phase polymer-NP-LC medium was treated by the Bruggeman method, considering the effective permittivity obtained for the NP-LC medium and the permittivity of the NP-polymer medium.

Numerical simulations were performed in high frequency mode, in typical intervals of variation of the permittivity of the constituents.

For the PDLC system, the effective permittivity was obtained and the differences between the two models were discussed. The results are in agreement with the experiments [25]. The modulus of the difference between the Maxwell Garnet effective permittivity and the Bruggeman one increases with the LC fraction, at constant constituents’ permittivity.

In the case of NPs-doped LCs, the difference between the two methods is modest at low NP concentrations (0.0001–0.001), increasing in importance at higher values. If the NP permittivity is equal to that of the LC host, the volume fraction does not influence the calculated effective permittivity.

Using MG and Bruggeman models, the effective permittivity of the NPs-doped PDLCs was simulated, obtaining information on its dependency on NPs permittivity and volume fractions.

The developed method can be applied for the calculation of the effective permittivity in the case of composites of LCs with quasi-spherical NPs such as titanium dioxide and zinc oxide. In the case of a composite in which one of the phases has an unknown permittivity, the models can be applied to extract it from the measured composite permittivity. The method also retains a general character and can be applied to other similar multiphase composites.

## Figures and Tables

**Table 1:** Comparative presentation of calculated PDLC film permittivity using Maxwell Garnett (Equation (10)) and Bruggeman (Equation (14)) formulas, and experimental results [25]; the field permittivity measured at low electric field corresponds to $\varepsilon_{⊥}$ of the LC and at high electric field to the $\varepsilon_{II}$ of the LC (LC aligned in the direction of the field).

Nematic Liquid Crystal	LC Permittivity	Calculated Film Permittivity Maxwell Garnett ^1^, Equation (9)	Calculated Film Permittivity Bruggeman ^1^, Equation (13)	Measured Film Permittivity [25]
E7	$\varepsilon_{⊥}$ 5.2	6.5	6.5	6.7
	$\varepsilon_{II}$ 19.0	12.5	12.7	14.5
	$\varepsilon_{LC,random}$ 9.8	8.9	8.8	-
ZLI 1840	$\varepsilon_{⊥}$ 4.3	6.0	5.9	5.7
	$\varepsilon_{II}$ 16.2	11.5	11.6	12.6
	$\varepsilon_{LC,random}$ 8.3	8.2	8.2	-

^1^ The calculated values are rounded in accordance with LC permittivity values.

## Sample Availability

Not available.
